# The use of k-t PCA accelerated dual-venc 3D flow MRI to assess hemodynamics before and after flow diverting stent implantation in cerebral aneurysm models

**DOI:** 10.1186/1532-429X-16-S1-W29

**Published:** 2014-01-16

**Authors:** Daniel Giese, Christoph Kabbasch, Dennis Hedderich, David Maintz, Thomas Liebig, Alexander Bunck

**Affiliations:** 1 Radiology, University Hospital Cologne, Cologne, Germany

## Background

Hemodynamic parameters are known to play a crucial role in the growth and rupture of intracranial aneurysms [Appanaboyina IntJNumerMethodsFluids2008]. Flow diverting stents have emerged as an alternative to coiling by using fine meshes to divert flow away from the aneurysm sac to promote thrombus formation and remodeling of the aneurysm while keeping side branches and perforating arteries patent. However, the post procedural course of these aneurysms is not fully predictable. Time-resolved 3D phase-contrast MRI allows for the assessment of three-directional velocity fields. Due to intrinsic long acquisition times, a single velocity encoding step (venc) is typically used [Acevedo-Bolton JCMR2013]. In this work, we use k-t Principal Component Analysis (PCA) [Pedersen MRM2009] in combination with a dual-venc acquisition to acquire highly undersampled 3D flow data for improved accuracy of lower velocities. Flow patterns within the aneurysms are analysed for different stent sizes, including optimal and oversized stents, and compared to the native models.

## Methods

3D digital subtraction angiography data were used to build true to scale silicon models of two different cerebral aneurysms. Model 1 was duplicated four times: one replica was left unstented (native), a 4 mm (size of parent vessel), 5 mm and 6 mm diameter flow diverting stent was deployed in the three remaining replicas. Model 2 was duplicated three times: one replica was left unstented (native), a 4 mm (size of parent vessel) and 5 mm flow diverting stent was deployed in the two remaining replicas. Connected to a pulsatile flow pump, undersampled (factor: 6-8) dual-venc time-resolved 3D phase-contrast MRI data were acquired in all models (1 × 1 × 1 mm3 spatial resolution, 42 ms temporal resolution, low venc: 15/25 cm/s, high venc: 100/150 cm/s). Dual-venc data were reconstructed using k-t PCA in combination with an un-aliasing algorithm [Lee MRM1995]. Particle traces were analysed.

## Results

**Figure 1:**
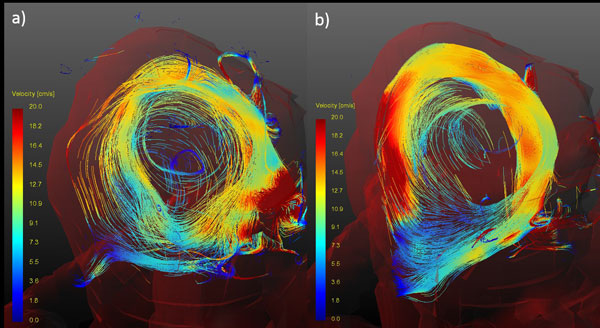
**Particle traces of a single-venc (a) and dual-venc (b) acquisition inside a stented aneurysm model**.

**Figure 2:**
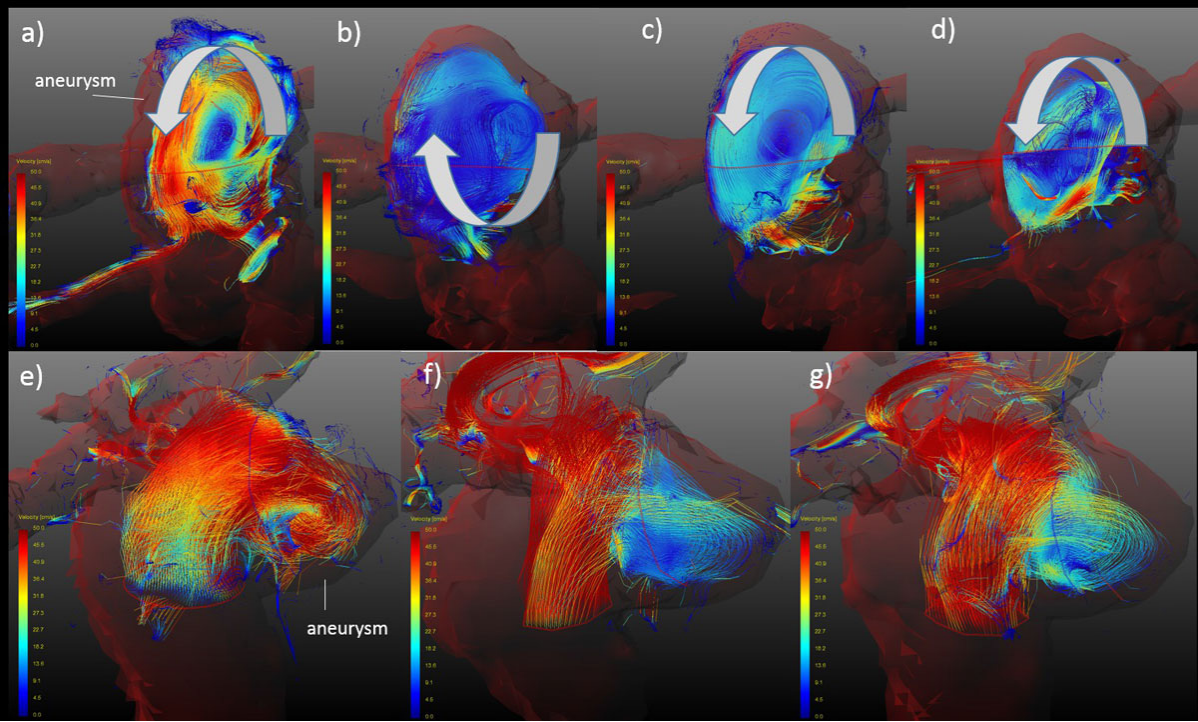
**Particle traces in both aneurysm models (Model1: a-d, Model2: e-g)**. a) and e) correspond to the unstented (native) models, b) and f) were stented with a 4 mm, c) and g) with a 5 mm and d) with a 6 mm diameter flow diverter. The main flow direction is depicted with arrows. Note the inverted flow direction in b) compared to a), c) and d).

Improved flow visualization by the dual-venc method as compared to a single-venc acquisition is shown in Figure 1. Flow in the stented aneurysms showed severely different behavior as compared to the native models. Furthermore, strongly differing flow behavior between the models deployed with different stent sizes were observed (Figure 2).

## Conclusions

Using k-t PCA in combination with a dual-venc time-resolved 3D flow acquisition allows for the simultaneous assessment of flow inside the parent artery and the aneurysm. Strongly differing hemodynamic behaviors were observed between native and stented aneurysms emphasizing the difficulty of predicting post-interventional flow conditions. Furthermore, these preliminary results demonstrate the significance of stent sizing and its potential influence on flow behavior. Especially in complicated geometries, flow patterns which potentially influence flow diversion and thrombus formation can be measured.

